# Supplementary material for: PIK3C3 Inhibition Promotes Sensitivity to Colon Cancer Therapy by Inhibiting Cancer Stem Cells
Source: Cancers (Basel). 2021 Apr 30;13(9):2168. doi: 10.3390/cancers13092168 (PMC8124755; doi:10.3390/cancers13092168)
Supplement: Supplementary file 1 [file cancers-13-02168-s001.zip › cancers-1187660-suppl-final.pdf]

cancers

MDPI

*Supplementary Materials*

# PIK3C3 Inhibition Promotes Sensitivity to Colon Cancer Therapy by Inhibiting Cancer Stem Cells

**Balawant Kumar, Rizwan Ahmad, Swagat Sharma, Saiprasad Gowrikumar, Mark Primeaux, Sandeep Rana, Amarnath Natarajan, David Oupicky, Corey R. Hopkins, Punita Dhawan and Amar B. Singh**

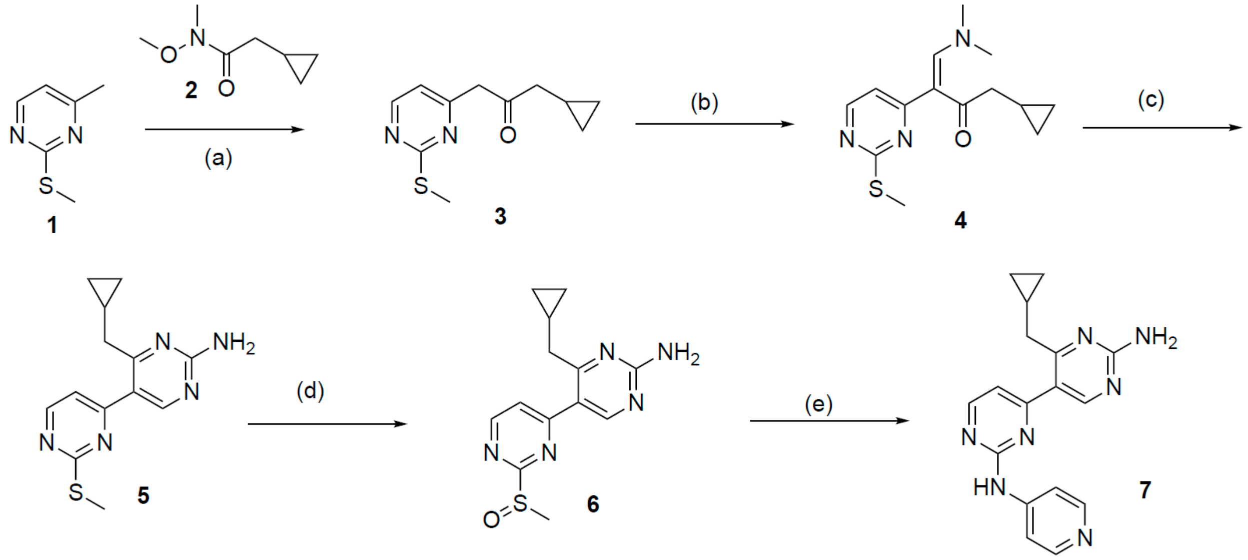


**Figure S1.** Sheme-1 of the synthesis of 36-077: Scheme-1, Reagents and conditions. (**a**) LDA (2.0 M in THF),THF, 2,-10oC; (**b**) DMF-DMA,80oC, 2 h, 80%; (**c**) N-methylguanine,K2Co3 DMF, 120oC,3h,rt,16hr, 72%; (**d**) m-CPBA,CH2Cl2, 10oC,45 mins,67%; (**e**) LiHMDS,4-aminopropyridine,THF,-10oC- > 1.5 h ,6%.

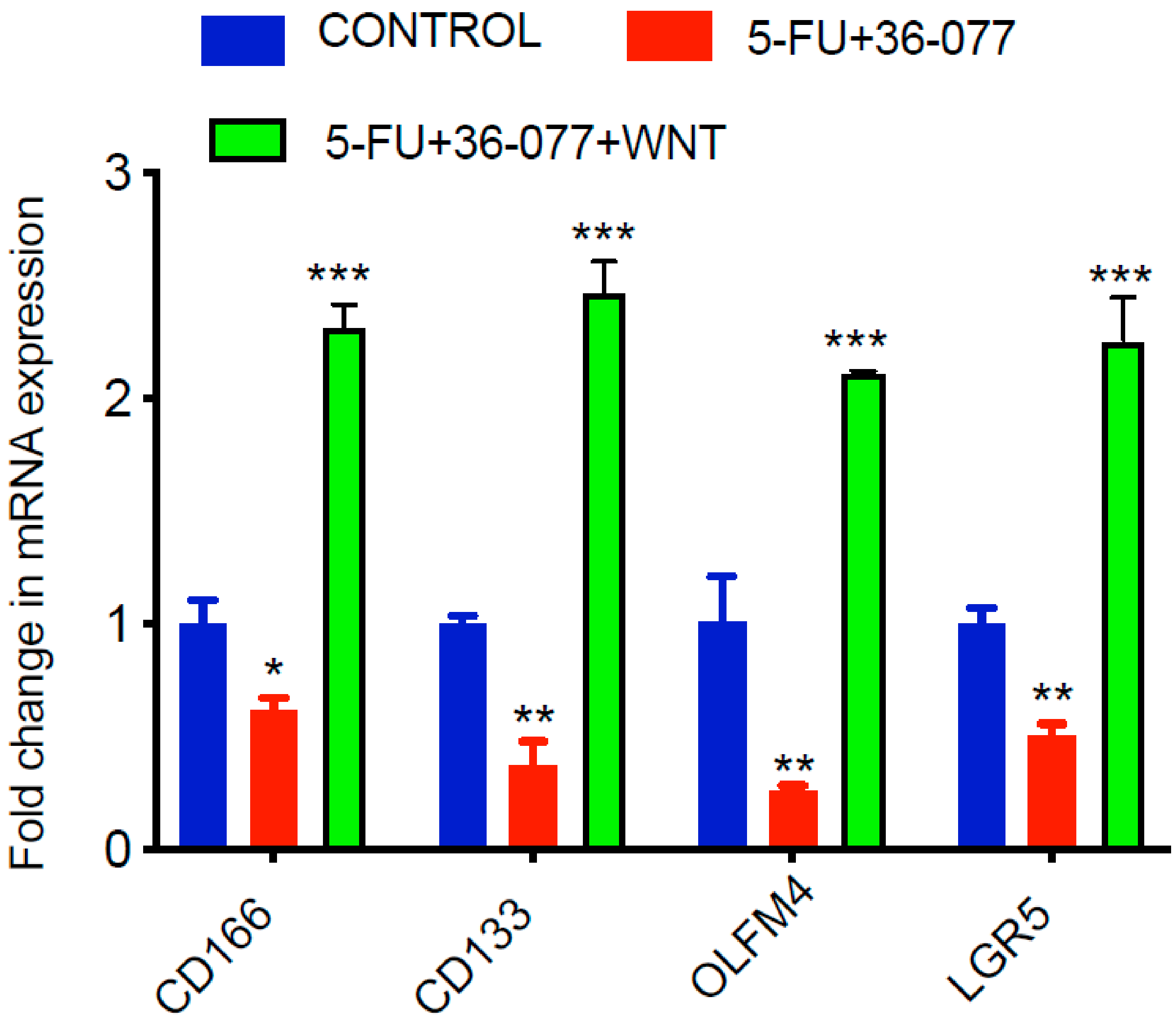


**Figure S2.** Wnt-signaling activation protects cancer stem cells from the co-treatment effects of 5-FU and 36-077: Real time qPCR for expression of stem cells markers using total RNA isolated from the HCT116 cells treated with 5-FU and 36-077 in combination with or without WNT3A. Data is presented as mean+sem. Statistical significance was determined by 1-way ANOVA and post hoc Tukey's test for pairwise comparison. *** $p < 0.001$, ** $p < 0.01$, * $p < 0.05$.

**Table S1.** List of reagents, antibodies and kits.

| S.N. | Antibody/Kit | Company | Catalog Number |
|---|---|---|---|
| 1 | jetPRIME transfection Reagent | Polyplus transfection | 114-07 |
| 2 | Cleaved caspase 3 rabbit antibody | Cell signaling Technology | 9661S |
| 3 | Anti-LC3 rabbit antibody | Sigma | L8918-25UL |
| 4 | Anti-p62/SQSTM1 rabbit antibody | Sigma | P0067-25UL |
| 5 | β-Actin mouse antibody | Santa Cruz technology | sc-47778 |
| 6 | Cyclin D1 Monoclonal rabbit antibody | Cell signaling Technology | 55506 |
| 7 | Corning Matrigel Matrix | Corning | 356255 |
| 8 | β-Catenin mouse monoclonal antibody | BD Biosciences | 610153 |
| 9 | Phospho-β-Catenin (Ser33/37) monoclonal rabbit antibody | Cell signaling Technology | 2009S |

| | | | |
|---|---|---|---|
| 10 | GSK-3β (D5C5Z) rabbit monoclonal antibody | Cell signaling Technology | 12456 |
| 11 | Phospho-GSK-3β (Ser9) (D85E12) monoclonal rabbit antibody | Cell signaling Technology | 5558 |
| 12 | Survivin (71G4B7) Rabbit monoclonal antibody | Cell signaling Technology | 2808 |
| 13 | OLFM4 (D1E4M) XP Rabbit monoclonal antibody | Cell signaling Technology | 14369 |
| 14 | Dual Luciferase assay kit | Promega | E2920 |

**Table S2.** Stem cells maker primer.

| **S.N** | **Primer** | **5′---------------Sequence---------------3′** |
|---|---|---|
| 1 | H_ β-ACTIN FP | CCA AGC ACA ATG AAG ATC AA |
| 2 | H_ β-ACTIN RP | ACA TCT GCT GGA AGG TGG AC |
| 3 | H_OLFM4FP | TGC CAT TCG CCG AGA AAT C |
| 4 | H_OLFM4RP | GGA CGA CAG GGG TGT TTT GAT |
| 5 | H_CD133FP | GGT GCT GTT CAT GTT CTC CA |
| 6 | H_CD133FP | ACC GAC TGA GAC CCA ACA TC |
| 7 | H_LGR5FP | CTC CCA GGT CTG GTG TGT TG |
| 8 | H_LGR5RP | GCT CGC AAT GAC AGT GTG TG |
| 9 | H_CD166FP | TAG CAG GAA TGC AAC TGT GG |
| 10 | H_CD166RP | CGC AGA CTA GTT CCA GCA |
| 11 | M_ β-ACTIN FP | CCA GAG CAA GAG AGG TAT CC |
| 12 | M_ β-ACTIN RP | CTG TGG TGG TGA AGC TGT AG |
| 13 | M_CD133FP | GAA AAG TTG CTC TGC GAA CC |
| 14 | M_CD133RP | TCT CAA GCT GAA AAG CAG CA |
| 15 | M_CD166FP | TTTGGCGCAGATGGAACTGA |
| 16 | M_CD166RP | TGTGCGTTGAAAGGAGGTG |
| 17 | M_LGR5FP | CTA CTT GAC TTT GAG GAA GAC C |
| 18 | M_LGR5RP | AGG AAA GCG CCA GTA CTG C |
