# Supplementary material for: PIK3C3 Inhibition Promotes Sensitivity to Colon Cancer Therapy by Inhibiting Cancer Stem Cells
Source: Cancers (Basel). 2021 Apr 30;13(9):2168. doi: 10.3390/cancers13092168 (PMC8124755; doi:10.3390/cancers13092168)

Raw Data of Figure 1

C.

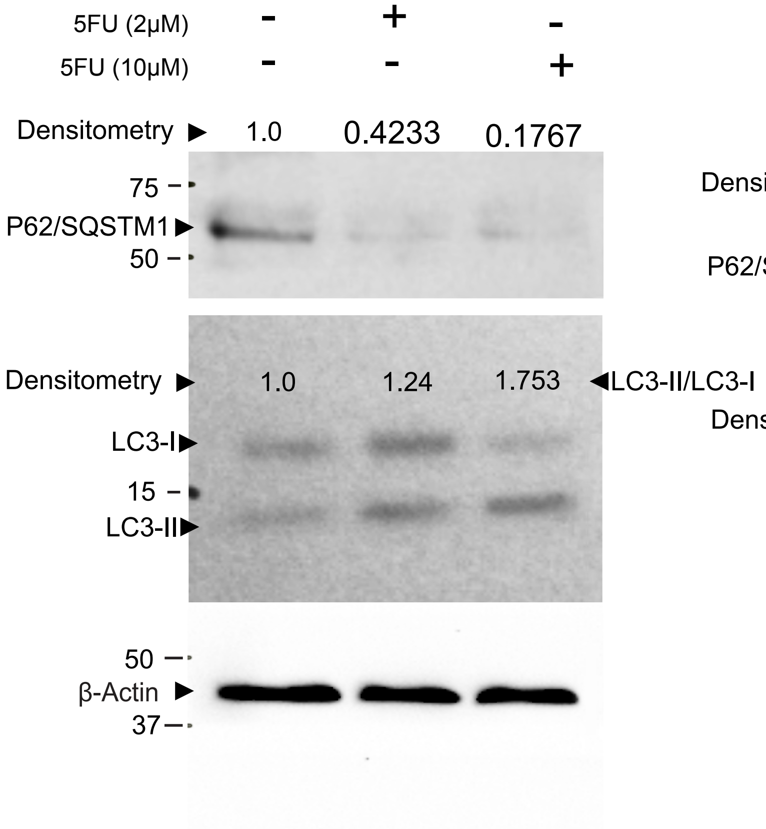

E.

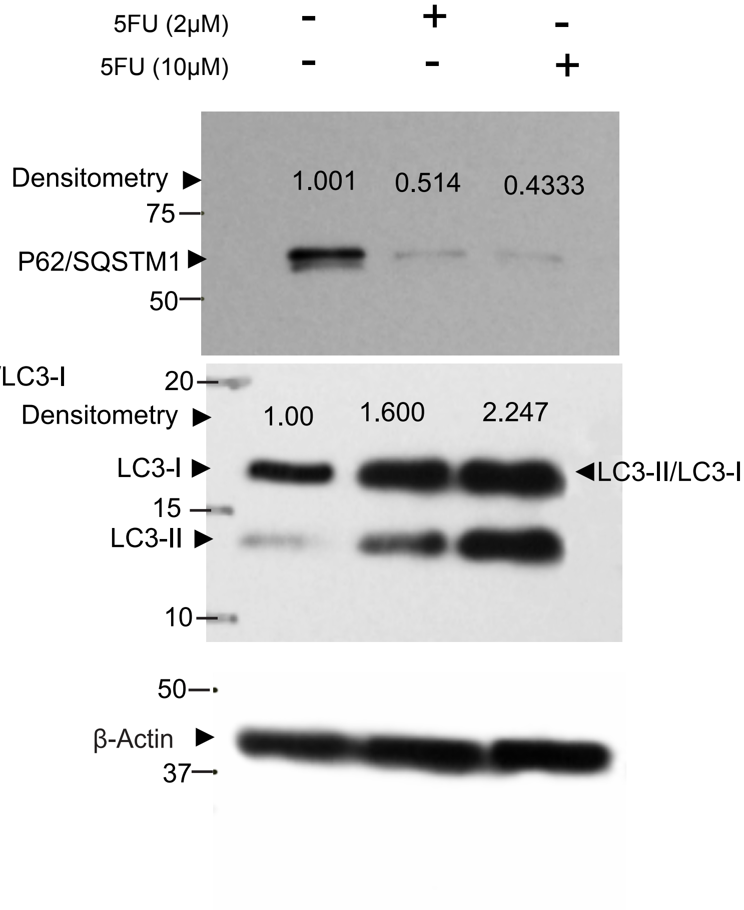

G.

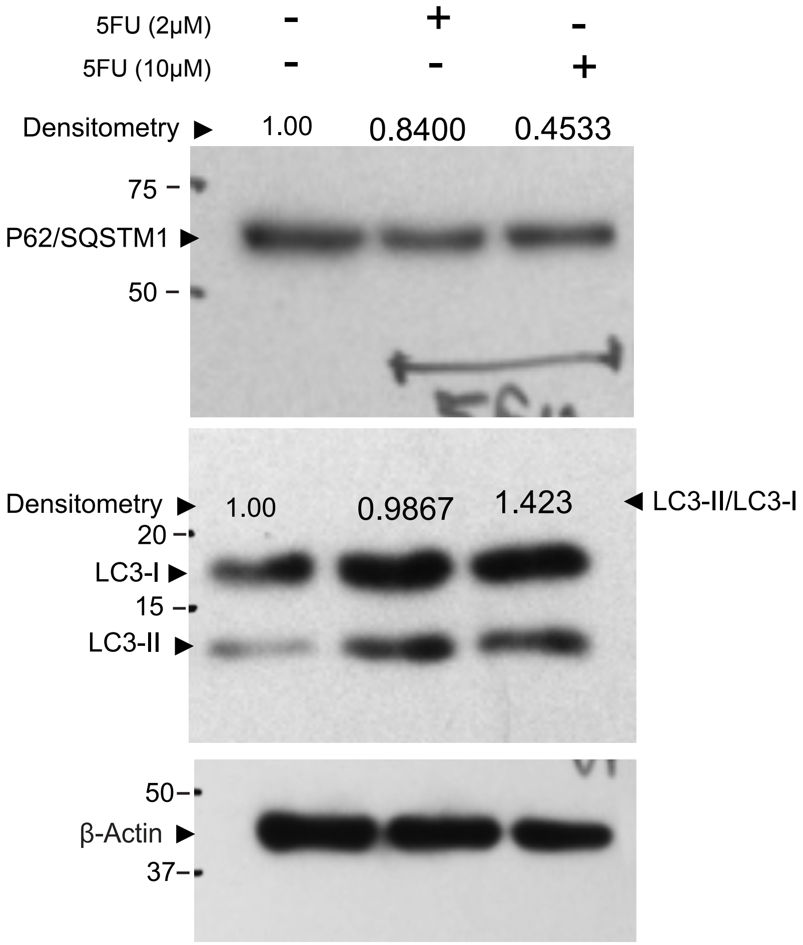

Raw Data of Figure 3

C.

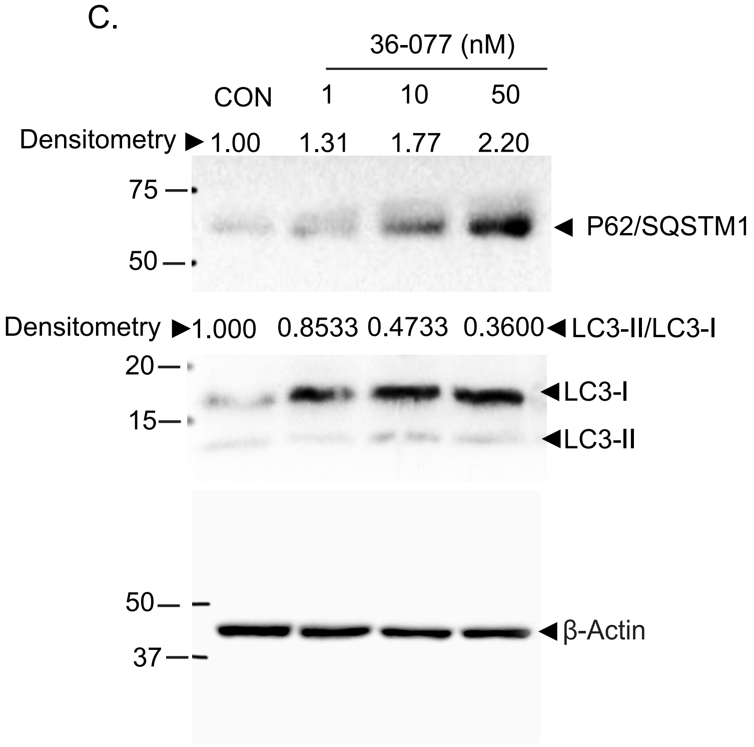

F.

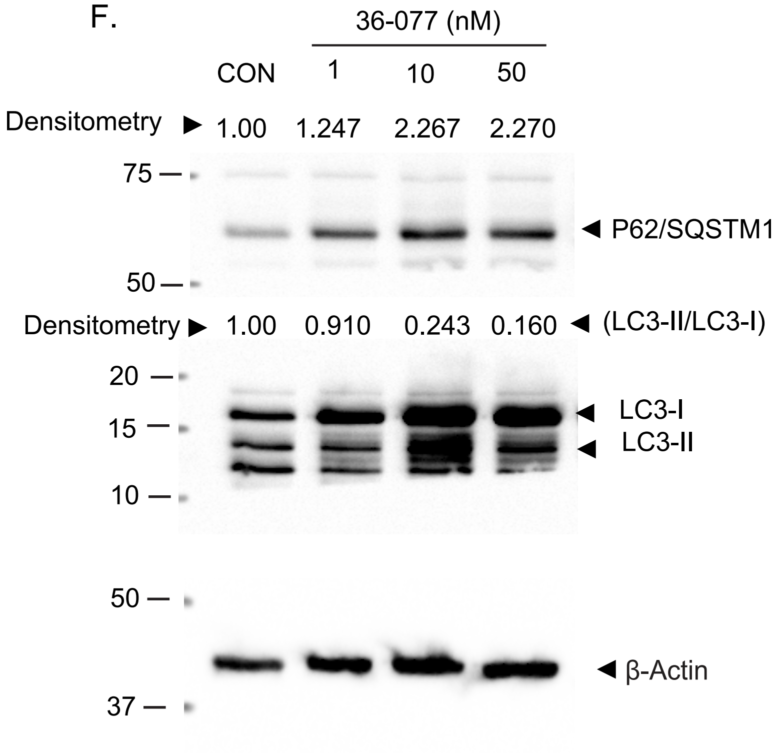

Raw Data of Figure 4

B.

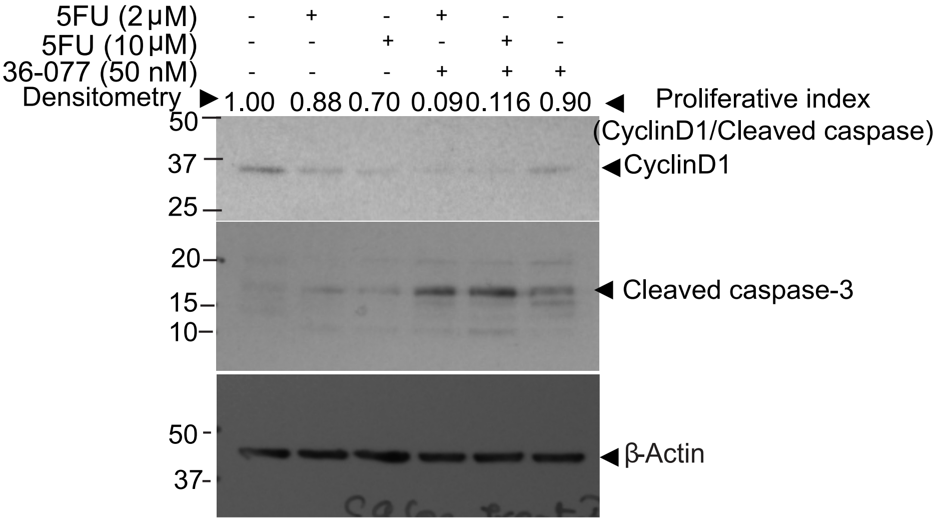

D.

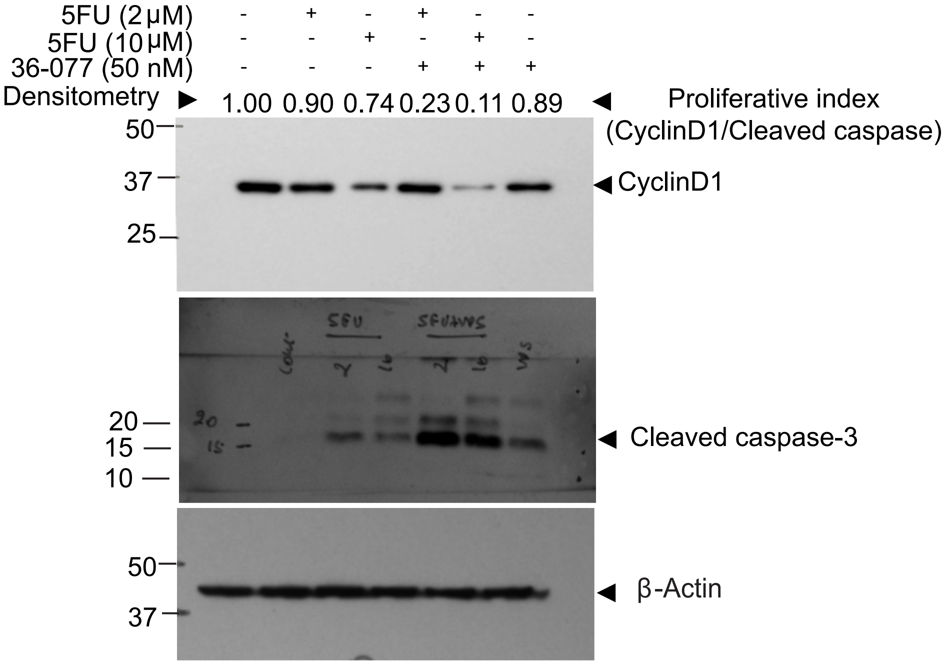

F.

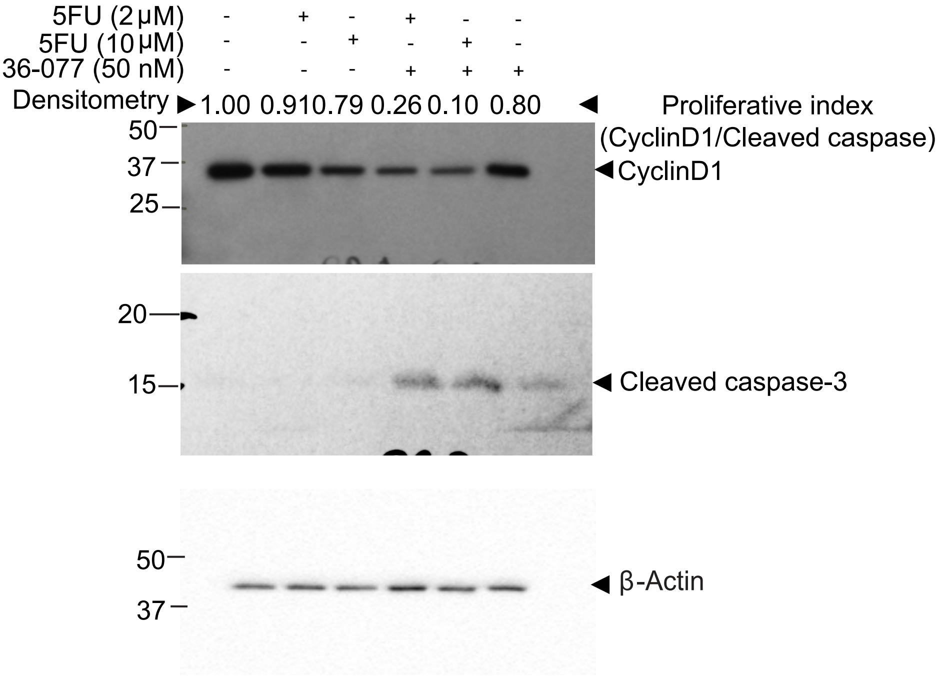

Raw data of figure 5

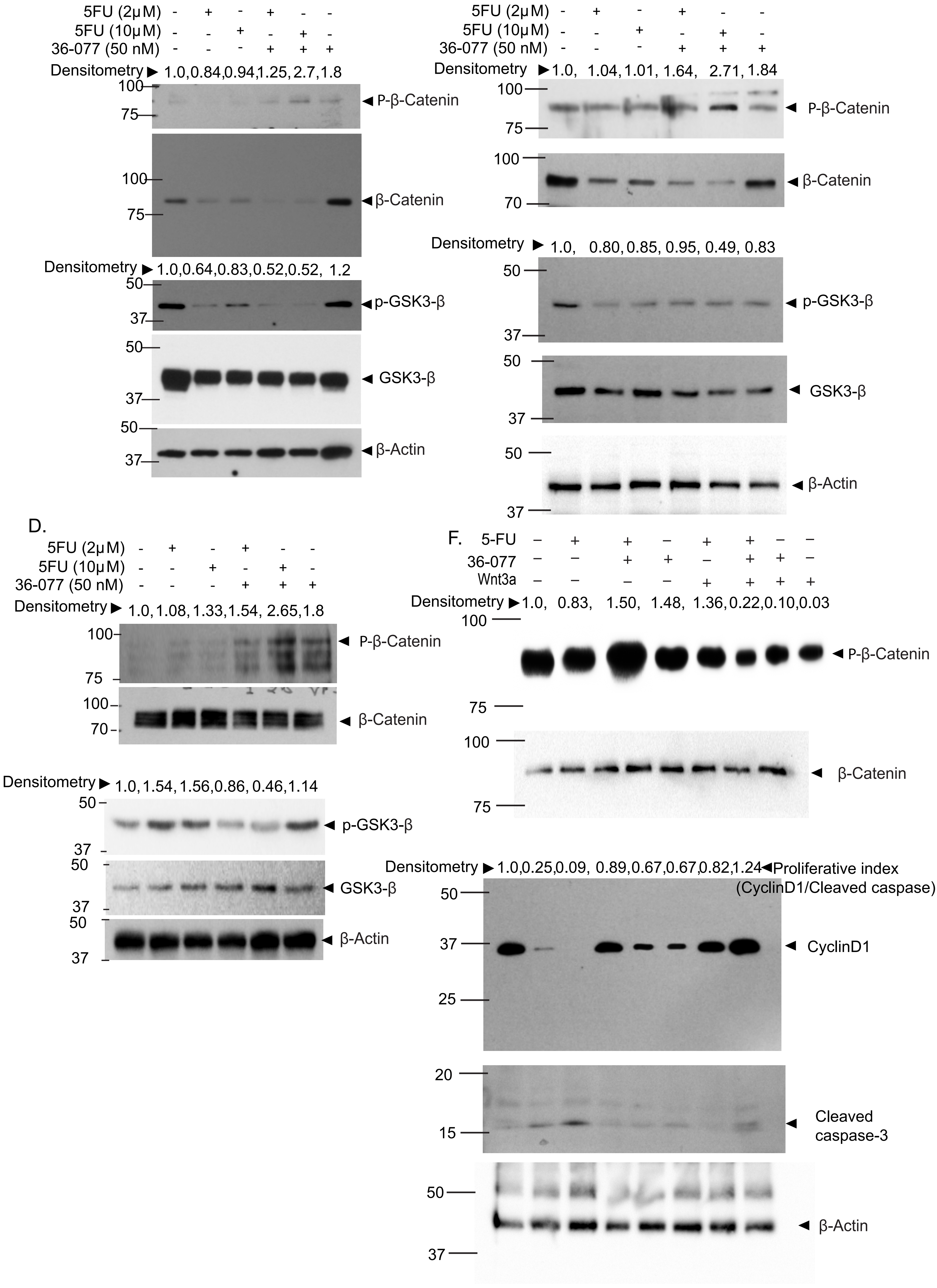

Raw data of figure 6

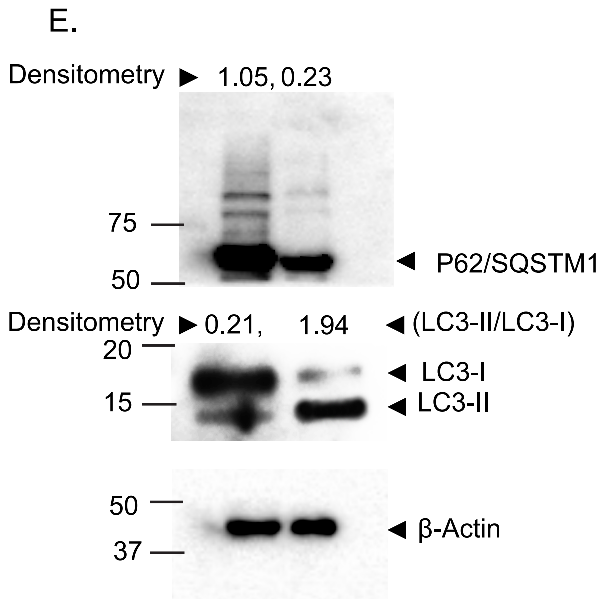

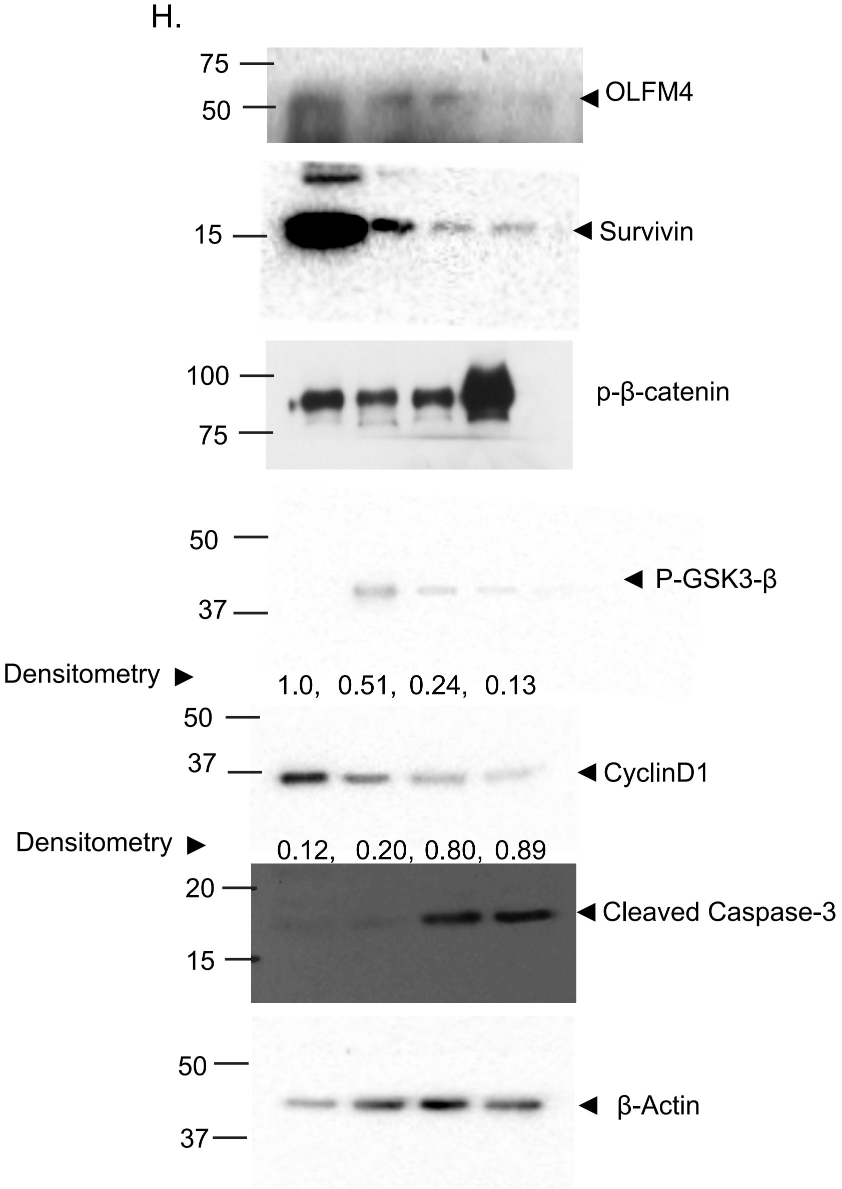

Supplement: Supplementary file 1 [file cancers-13-02168-s001.zip › cancers-1187660-WB-proof.pdf]
